# Smart Wash: Accelerated Membrane Washing Method in Immunoblot

**DOI:** 10.1002/elps.8104

**Published:** 2025-04-17

**Authors:** Ethan P. Stevenson, Christopher K. Schroeder, Richard Chan, Herbert M. Geller, Yasuhiro Katagiri

**Affiliations:** ^1^ Developmental Neurobiology Laboratory Cell and Developmental Biology Center National Heart, Lung, and Blood Institute, National Institutes of Health Bethesda Maryland USA; ^2^ Embi Tec San Diego California USA

**Keywords:** chemiluminescenc, fluorescence, immunoblot, membrane washing, western blot

## Abstract

Immunoblot, also known as western blot, is a well‐established procedure in life science. It is commonly used to determine the relative size and abundance of specific proteins, as well as posttranslational modifications of proteins. While this method is widely employed due to its simplicity, it can take hours or even days to complete. Despite considerable efforts to reduce the overall procedure time, particularly for antibody incubation, the steps involving membrane rinsing have remained unchanged since the development of the immunoblot technique. In this context, we introduce an innovative device called the “Smart Wash,” designed to significantly reduce the washing intervals by utilizing a motorized salad spinner. The principle of Smart Wash is akin to that of a household washing machine: the container holds the membranes during the rinsing cycle, and the basket moves the membranes along with the washing solution in the container. We have optimized the rinsing conditions, including the volume of the washing solution, rotation speed, number of washing cycles, and direction. This straightforward device empowers researchers to significantly enhance the efficiency and productivity of immunoblotting analysis.

**Abbreviations**
AP: alkaline phosphatase
CDR: cyclic draining and replenishing
CGS‐1: Can Get Signal‐1 Immunoenhancer Solution
CGS‐2: Can Get Signal‐2 Immunoenhancer Solution
PBS‐T: phosphate buffer saline containing 0.1% Tween20

## 1 Introduction

Immunoblot, also known as western blot, is crucial in a wide range of scientific and clinical research. This technique assesses the abundance and relative size of proteins and their posttranslational modifications [1, 2, 3, 4, 5, 6]. Immunoblot involves a series of incubations with different immunochemical reagents, interspersed with membrane rinsing steps. While each step is vital for the accurate identification and quantification of proteins of interest, rinsing is essential to eliminate unbound reagents and minimize background, thereby enhancing the signal‐to‐noise (S/N) ratio. Insufficient rinsing may lead to an elevated background, resulting in a lower S/N ratio. Although immunoblot yields valuable insights into academic research, diagnostics, and therapeutic testing, it is a labor‐intensive and time‐consuming process. Each iteration can take hours or even days to complete, making these assays more time‐consuming than other advanced techniques. To address these limitations, the procedure has evolved, with subsequent innovations enhancing sensitivity, speed, and quantification [7, 8, 9, 10, 11, 12, 13, 14]. New instruments such as the Bandmate Automated Western Blot Processor, SNAP i.d. 2.0 Protein Detection System, and iBind Automated Western Systems have also been introduced. Although many reports detail improved methods for membrane incubation with various probes during immunoblot, significant advancements in membrane rinsing steps remain scarce. A common approach involves placing the membrane in a container with enough PBS‐Tween 20 to fully submerge it and agitating it (typically for 10 min, repeated three times). This process takes at least 30 min after both primary and secondary antibody incubations. In our previous reports [15, 16], we employed a commercial salad spinner to expedite membrane rinsing within a short period (∼5 min), but the conditions were not fully optimized. Additionally, we observed variable image quality when using a manual salad spinner, depending on the rotation speed, and the operator conducting the experiment. In the present study, we introduce a motorized salad spinner named “Smart Wash” and optimize the rinsing conditions. The Smart Wash not only accelerates the rinsing process (approximately 3 min after antibody incubation instead of 30 min) but also produces consistent results and minimizes operator errors.

## 2 Materials and Methods

### 2.1 Assembly of the “Smart Wash” Device

**FIGURE 1:**
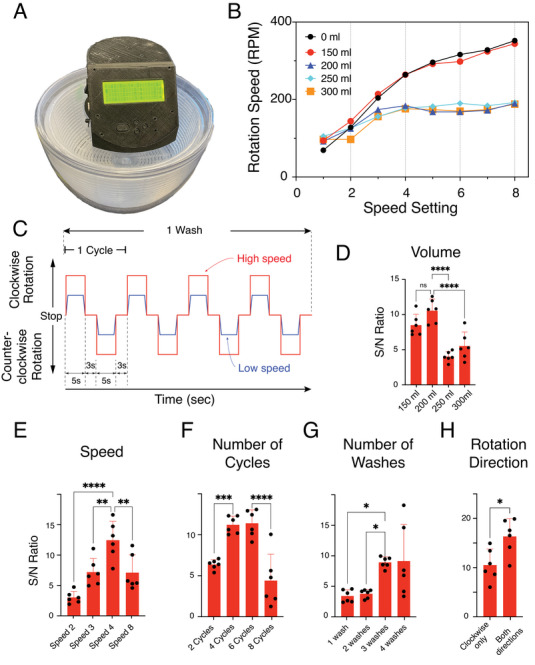
(A) Photograph of Smart Wash device. (B) Rotation speed of the basket with and without PBS‐T. The presence of more than 200 mL of PBS‐T reduced the speed. (C) Schematic diagram of Smart Wash steps. (D–H) The effects of various rinsing conditions on the S/N ratio. The standard condition is 200 mL of PBS‐T, speed setting #4, four cycles per wash, three washes, and both clockwise and counterclockwise rotations. (D) Different volumes of PBS‐T. (E) Different speed settings. (F) Different numbers of cycles per wash. (G) Different numbers of washes. (H) Rotation directions. Source images and fluorescence measurements are shwon in Figures S1 and S2. ****p < 0.0001, ***p < 0.001, **p < 0.005, *p < 0.05.

We developed a system to motorize and control the OXO Salad Spinner (container dimensions: 26.6 × 26.6 × 15.2 cm, SKU: 32480V2B). The basket is rotated by a battery‐powered gear motor assembly (Walfront, 25GA‐370 DC 12 V Micro Gear Box Motor) in either the clockwise or counterclockwise direction at a maximum speed of 350 rpm without any solution (Figure 1A,B). The control software was designed to provide the necessary hardware controls and a user interface with multiple screens that convey status information. This allows protocols to be edited, stored, and retrieved.

### 2.2 Antigens and Antibodies

The conditioned media containing the secreted (His)_6_‐tagged alkaline phosphatase (AP‐His, GenHunter) from transfected HEK‐293 cells were used for this study [16]. pEGFP was also transfected into HEK‐293 cells. HEK‐293 cell lysates prepared with RIPA buffer (Thermo Fisher Scientific) in the presence of a protease inhibitor cocktail (Calbiochem) were used as well [16]. The antibodies used in this study, including the dilution information, are listed in the Supporting Table.

### 2.3 SDS‐PAGE, Electrophoretic Transfer, Slot Blot, Antibody Incubation With Membranes, and Image Acquisition

These procedures were performed as previously described [15, 16]. Unless otherwise stated, PVDF membranes were used in this study. Membranes for slot blot were prepared with a Hoefer PR648 and carried both secreted AP‐His and control culture media containing 10 % Fetal Bovine Serum (120 µL/slot, 6 slots each, Figure S1A). For antibody incubation, 10 % Can Get Signal reagent‐1 and ‐2 (TOYOBO) were used for primary and secondary antibodies, respectively, employing the cyclic draining, and replenishing (CDR) method [15, 16]. (Detailed information is in the Supporting Table. Bound antibodies were visualized for both fluorescent and chemiluminescent detections using an Azure c600 Imaging System (Azure Biosystems). For chemiluminescent detection, the LumiGLO Peroxidase Chemiluminescent Substrate Kit (SeraCare) was used.

### 2.4 Membrane Washing

#### 2.4.1 Conventional Washing

After antibody incubation, the membranes were thoroughly rinsed with deionized water to remove most of the unbound antibodies until the detergent‐related bubbles disappear (Movie S1). Subsequently, they were rinsed with PBS‐T (50 mL) in a container (12.5 × 10 cm) with agitation for 10 min, three times after primary antibody incubation, and for 5 min, six times after secondary antibody incubation. The total volume of PBS‐T required for one membrane was 450 mL.

#### 2.4.2 Smart Washing

Membranes were extensively rinsed with deionized water as described above (Movie S1), followed by a quick but thorough rinse under the basket using our Smart Wash system with PBS‐T under various conditions (Movies S2 and S3). One rinse consists of multiple washes in the Smart Wash system. Figure 1C illustrates the detailed operation of the unit. A detailed procedure for the Smart Wash is provided in the Supporting Information.

### 2.5 Quantitative Measurement

Sequential raw 16‐bit TIF images obtained from the slot blot were analyzed by Image Studio Lite (Ver. 5.2.5, LI‐COR Biosciences). To prevent saturated signals, we plotted the average and standard deviation of relative units (RU) derived from each of the six samples. All statistical analyses were carried out in GraphPad Prism (Ver. 10.3.1, GraphPad Software).

## 3 Results and Discussion

Rinsing steps are essential for removing unbound reagents that may interfere with the detection of target molecules and help reduce the background signals. Improving the S/N ratio can enhance assay sensitivity. Variability in the rinsing conditions such as rinsing duration and volume usage could be responsible for the inconsistency of the results observed with other blotting protocols. Thus, optimizing rinsing steps is key to a successful immunoblot. Since the Smart Wash is a new device, the optimal rinsing conditions necessary to enhance the S/N ratio should be determined empirically.

Figure 1C shows the details of Smart Wash steps: one cycle consists of (1) clockwise rotation for 5 s, (2) pause for 3 s, (3) counterclockwise rotation for 5 s, and (4) pause for 3 s. We tested various parameters, including the volume of washing solution per wash, rotation speed, number of cycles, number of washes, and rotation directions (simple direction or a combination of clockwise and counterclockwise directions). To quantitatively assess these factors, we employed a slot blot technique using conditioned media with fluorescent detection, chosen for its superior quantification capabilities, and wide dynamic range. After capturing images of multiple membranes under different rinsing conditions, we measured the signal strength for AP‐His and control media and calculated the S/N ratio (Figure 1D–H, Figures S1 and S2).

Initially, we characterized the basket rotation speed under various conditions (Figure 1B and Figure S1B). A nearly linear increase in the basket rotation speed was observed up to the speed setting #4 in the absence of PBS‐T, with a reduced increase beyond setting #4. A similar result was obtained when 150 mL of PBS‐T was used, where the bottom of the basket slightly touched the solution, resulting in minimal bubble generation (Figure S1B). Conversely, increasing volumes of PBS‐T reduced the rotation output, leveling off at 160 rpm beyond setting #3. This reduction is possibly due to the motor's insufficient power to maintain rotation in the presence of larger volumes of PBS‐T and fluid resistance, as well as the detergent‐related bubbles generated during the rotation (Figure S1B). Higher speed settings and greater volumes of PBS‐T created more bubbles.

The main principle of membrane rinsing in the immunoblotting procedure is passive diffusion. One might assume that using a larger volume of PBS‐T in the Smart Wash device would increase the S/N ratio. However, our findings indicate that the larger volumes actually reduced the S/N ratio (Figure 1D). Figure S1C shows that while signals on AP‐His slots were higher with 250 and 300 mL of PBS‐T, the control slots displayed higher signals compared to 150 and 200 mL, suggesting insufficient rinsing. Volumes of 150 or 200 mL of PBS‐T provided more efficient rinsing, thus resulting in a higher S/N ratio (Figure 1D and Figure S1C). This can be partially explained by the reduced rotation speed and increased bubble formation with larger volumes of PBS‐T as described above. Although detergents are commonly used in immunoblot, excessive bubbles can have a detrimental impact on the efficient rinsing of the membranes. This observation aligns with our finding that a speed setting of #4 produced lower signals on the control slots compared to the lower speed settings (#2 and #3), enhancing the S/N ratio (Figure 1E and Figure S1D).

**FIGURE 2:**
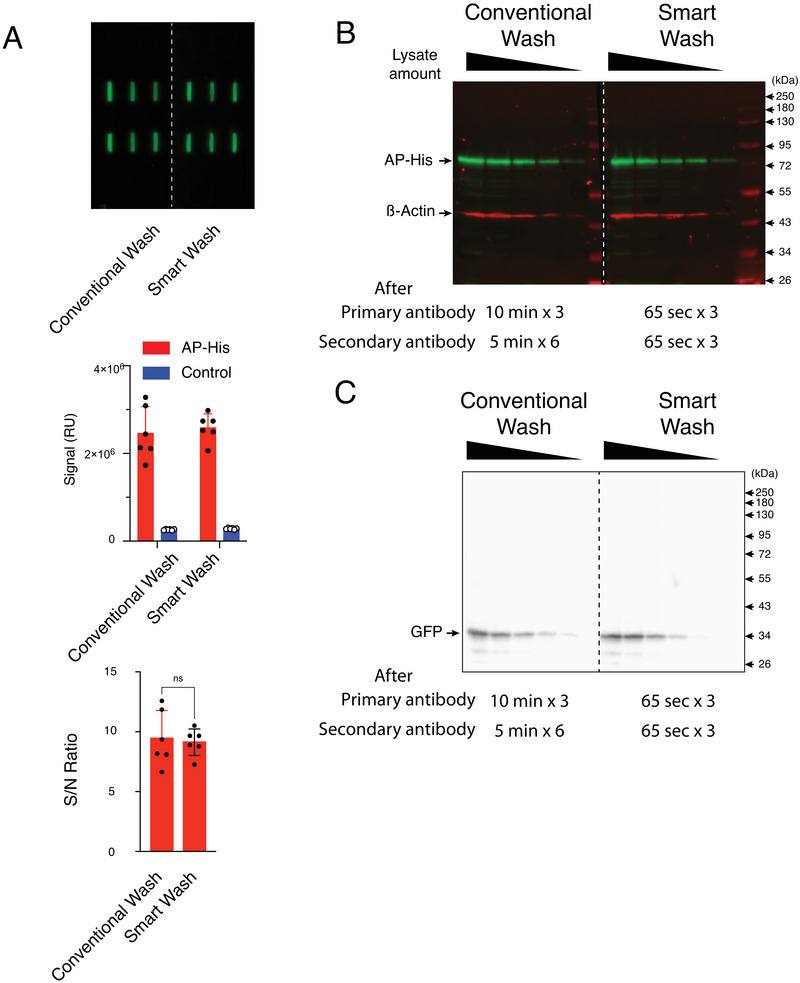
Comparison between conventional wash and Smart Wash (standard condition). (A) The signals for the secreted AP‐His from individual slots were measured and plotted, and the S/N ratio was calculated. (B and C) Different amounts of cell lysates (1:2 serial dilutions starting from 6 µg/lane; B: AP‐His‐transfected HEK293; C: GFP‐transfected HEK293) were separated by SDS‐PAGE, followed by immunoblot. (B) Simultaneous fluorescent detection with anti‐β‐actin and anti‐His‐tag antibodies. (C) Chemiluminescent detection with anti‐GFP antibody.

When immunoblot images show high background signals, it is common to increase rinsing times and volumes to reduce background signals, often along with performing additional buffer changes. However, rinsing too aggressively can sometimes diminish the desired signals. We observed such an effect (Figure 1F): eight cycles per wash (Figure S2A) and four washes per rinse (Figure S2B) reduced the signals on AP‐His slots. Conversely, insufficient rinsing increased the signals on the control slots, as seen with two cycles per wash (Figure S2A) and one or two washes per rinse (Figure S2B). Thus, careful optimization is crucial for sensitive detections. Simple clockwise rotation provided a sufficiently high S/N ratio with the anti‐His‐tag antibody but combining clockwise and counterclockwise rotations improved the S/N ratio (Figure 1H, Figure S2C). For the anti‐AP antibody, using both rotations also improved the S/N ratio by reducing control slot signals (Figure S2C). In summary, the optimal Smart Wash conditions include using 200 mL of PBS‐T, setting the speed to #4, performing four cycles per wash, and conducting three washes per rinse with bidirectional rotation in each cycle. This process requires a total of 1.5 L of PBS‐T. These optimized Smart Wash conditions produced results comparable to the conventional method in both signal strength and S/N ratio, as shown using slot blot (Figure 2A). The established protocol is presented in the Supporting Information. Each wash takes 65 s, totaling approximately 3 min, significantly reducing the overall rinsing duration compared to the 30 min required by the conventional washing method.

The semi‐automated Smart Wash device was designed to standardize and streamline the rinsing procedures in immunoblot. To illustrate the overall benefits, we applied the optimized Smart Wash condition to the PVDF membranes generated from SDS‐PAGE (Figure 2B,C). A comparison between the conventional wash and the Smart Wash shows comparable results in both fluorescent and chemiluminescent detections. Fluorescence immunoblot has the unique advantage of allowing multiple targets to be assayed on the same blot simultaneously, without the need to strip, and re‐probe. Smart Wash is efficient enough for the simultaneous detection of His‐tagged AP and β‐actin, with no apparent difference in the detection limits using the serial dilutions of cell lysates (Figure 2B). Chemiluminescent detection of the transfected GFP was quickly accomplished using Smart Wash, yielding results equivalent to those obtained with the conventional wash (Figure 2C). Additionally, Smart Wash was applied to nitrocellulose membranes (Figure S3). Slot blot analysis revealed similar S/N ratios between Smart Wash and conventional wash. The simultaneous detection of His‐tagged AP and β‐actin on the nitrocellulose membrane demonstrated the parallel results between two rinsing methods using serial dilutions of the cell lysates (Figure S3B). It should be noted that the transparent container of Smart Wash allows easy monitoring of membranes (Movie S3), and up to four membranes (8 × 6.5 cm) can be rinsed simultaneously without drastic changes in the quality of the images. No membrane tears were observed during the rinsing steps, indicating the gentle operations of Smart Wash. Fine‐tuning the rinsing conditions can lead to consistent and quantitative results, depending on the specific antibodies used in the study. We speculate that one reason for the accelerated rinse using Smart Wash is enhanced passive diffusion due to the higher volume of the solution. Additionally, the movement of the solution increases its efficiency, as shown in Figure 1D,E, resulting in a quick and efficient rinse of the membrane.

## 4 Concluding Remarks

Traditionally, the rinsing steps in immunoblots are labor‐intensive and time‐consuming. To overcome these challenges, we developed the motorized salad spinner, Smart Wash, which is as effective as traditional washing methods and applicable to both PVDF and nitrocellulose membranes for fluorescent and chemiluminescent detections. The advantages of Smart Wash include (1) increased speed (approximately 3 min after antibody incubation with Smart Wash instead of 30 min with conventional washing), (2) portability, (3) precise control of rinsing, (4) cost‐effectiveness compared to commercial automated instruments, and (5) reduced operator errors due to this precise control. Designed using a commercial salad spinner, the lid‐motor assembly (control unit) can be used with multiple containers, enabling the serial rinsing of membranes. Combining the CDR method [15, 16] with Smart Wash drastically reduces the overall time for the entire procedure, allowing for a much higher quantity of immunoblots per day when the blocked membranes are available. Thus, this simple device significantly boosts the efficiency and productivity of immunoblotting analysis.

## Author Contributions

Yasuhiro Katagiri and Herbert M. Geller conceived and designed the research. Richard Chan and Christopher K. Schroeder constructed the apparatus. Ethan P. Stevenson and Yasuhiro Katagiri performed experiments, analyzed data, and interpreted the results. Yasuhiro Katagiri, Ethan P. Stevenson, and Christopher K. Schroeder wrote the paper.

## Conflicts of Interest

Richard Chan is a founder of Embi Tec. Christopher K. Schroeder was an employee of Embi Tec. The remaining authors declare no competing interests.

## Supporting information



Supporting Information

Supporting Information

Supporting Information

Supporting Information

## Data Availability

The data that support the findings of this study are openly available upon request.
