# Supplementary material for: Smart Wash: Accelerated Membrane Washing Method in Immunoblot
Source: Electrophoresis. 2025 Apr 17;46(11-12):762–7. doi: 10.1002/elps.8104 (PMC12212287; doi:10.1002/elps.8104)
Supplement: Supplementary file 1 — Supporting Information [file ELPS-46--s001.pdf]

SUPPORTING INFORMATION:

Supporting Table

The antibodies used in this study, including their dilutions, are listed.

Antibodies used in this study

Antibody	Company	Catalog number
Mouse anti-ß actin antibody (1:2,000 dilution with 10 % CGS-1 solution)	Sigma	A5316
Rabbit anti-6X His tag antibody (1:2,000 dilution with 10 % CGS-1 solution)	Abcam	ab9108
Mouse monoclonal anti-GFP antibody (1:5,000 dilution with 10 % CGS-1 solution)	Proteintech	66002-1-g
Human Alkaline Phosphatase/ALPP Antibody (1:2,000 dilution with 10 % CGS-1 solution)	R&D Systems	MAB5905
ECL™ anti-mouse IgG, Horseradish peroxidase linked $F(ab')_2$ fragment from sheep (1:20,000 dilution with 10 % CGS-2 solution)	Cytiva	NA9310V
IRDye® 680RD Goat anti-Mouse IgG Secondary Antibody (1:5,000 dilution with 10 % CGS-2 solution)	LICOR	925-68070
IRDye® 800CW Goat anti-Rabbit IgG Secondary Antibody (1:5,000 dilution with 10 % CGS-2 solution)	LICOR	926-32211

Antibody incubation method

<table>
<tr><th>Antibody</th><th>Detection method</th><th>Incubation time (min)</th><th>Incubation Method</th><th>Incubation Temperature</th></tr>
<tr><td>Primary antibody</td><td rowspan="2">Fluorescent detection</td><td rowspan="2">20 min</td><td rowspan="2">CDR (6 rpm)</td><td rowspan="4">Room temperature</td></tr>
<tr><td>Secondary antibody</td></tr>
<tr><td>Primary antibody</td><td rowspan="2">Chemiluminescent detection</td><td rowspan="2">10 min</td><td rowspan="2">CDR (6 rpm)</td></tr>
<tr><td>Secondary antibody</td></tr>
</table>

Volume of antibody solution

Size of the membrane	Volume	Tube used
4 x 8 ~ 6.5 x 8 cm	8 ml	50 mL Conical Centrifuge Tubes
2 x 8 ~ 4 x 8 cm	3 ml	14ml Round-Bottom Polypropylene Tube

The implementation of the CDR incubation procedure on a hybridization oven can be seen at the following link (https://youtu.be/VYhLEIf3yeI).

Supporting Figure 1

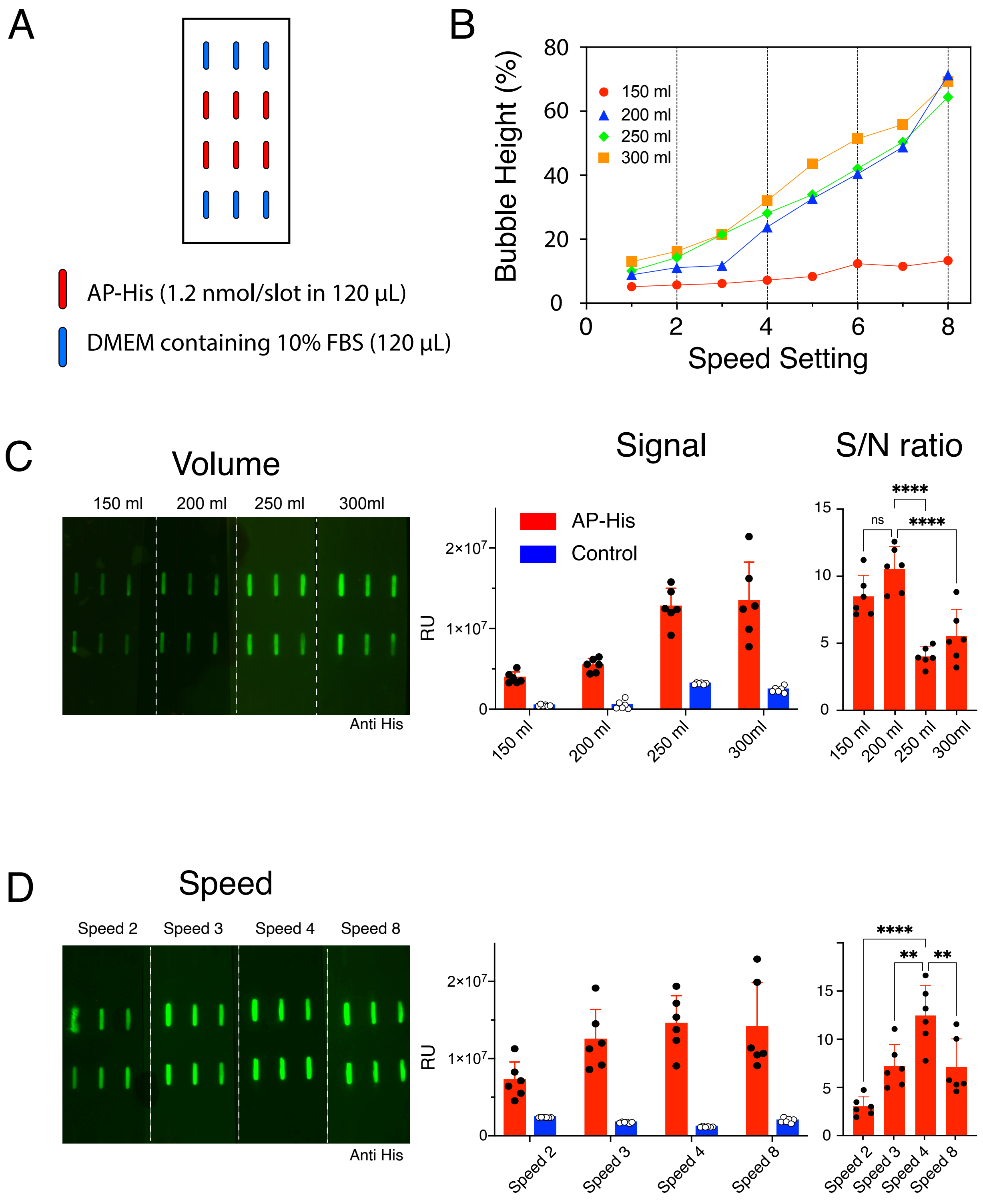

Supporting Figure 1

A. Configuration of slot blot. B. Relative bubble height during rotation. The peak height of bubbles is expressed as a percentage of the height of the Smart Wash container. C. Fluorescent image of 4 membranes rinsed in different volumes of PBS-T. Rabbit anti-6xHis tag and IRDye® 800CW Goat anti-rabbit antibodies were used for visualization. The signals from individual slots were measured, plotted, and the S/N ratio was calculated. D. Fluorescent image of 4 membranes rinsed at different speed settings. **** $P<0.0001$, ** $P<0.005$.

Supporting Figure 2

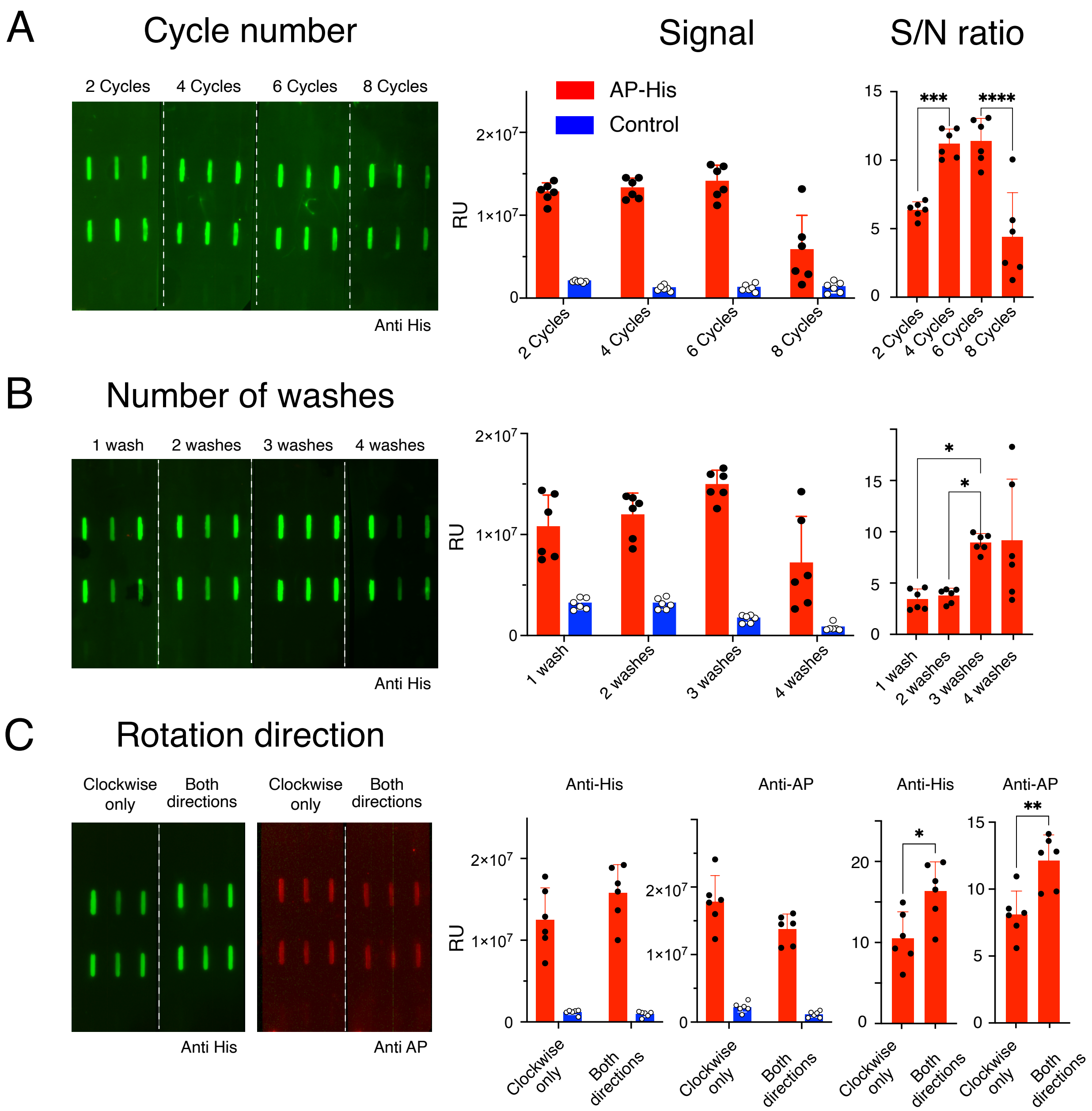

Supporting Figure 2

A. Fluorescent image of 4 membranes rinsed with different cycle numbers. The signals from individual slots were measured, plotted, and the S/N ratio was calculated. B. Fluorescent image of 4 membranes rinsed with different wash numbers. C. Fluorescent image of 4 membranes rinsed with different rotation directions. (Left) Rabbit anti-6xHis tag and IRDye® 800CW Goat anti-rabbit antibodies, and (right) Mouse anti-human alkaline phosphatase and IRDye® 680RD Goat anti-mouse antibodies were used. **** P<0.0001, *** P<0.001, ** P<0.005, * P<0.05.

Supporting Figure 3

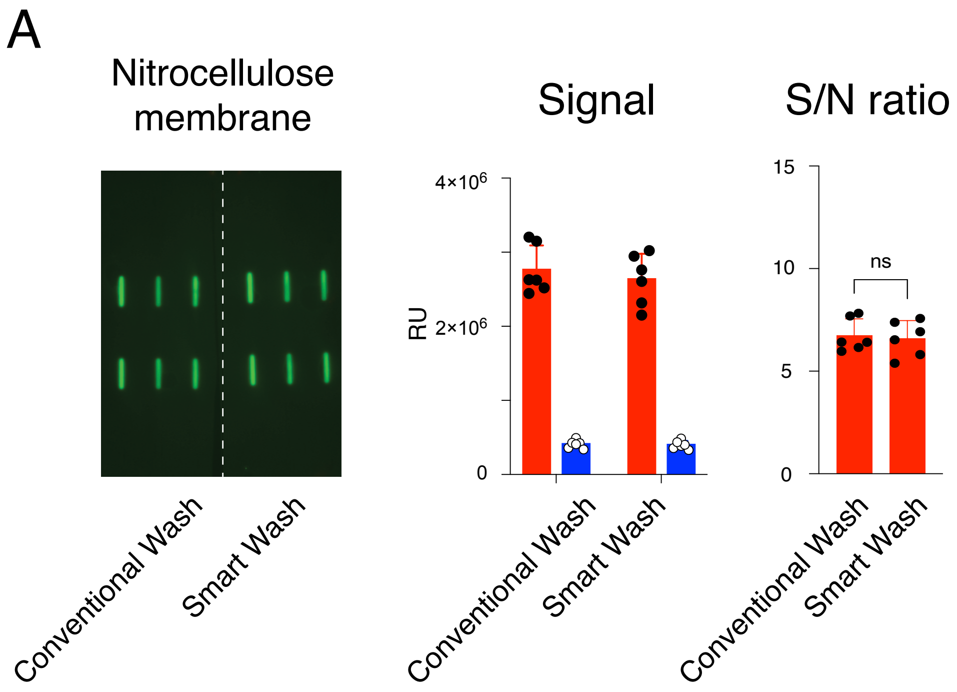

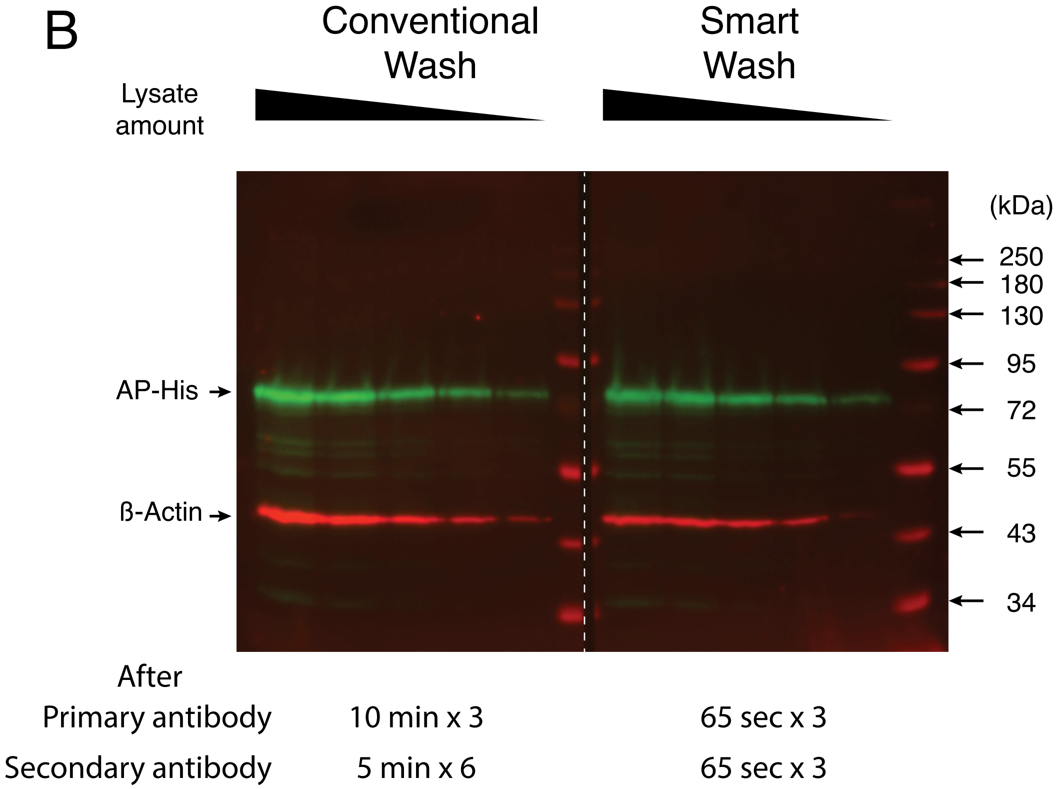

Supporting Figure 3

Comparison between Conventional Wash and Smart Wash (standard condition) using Nitrocellulose Membranes. A. Fluorescent image of two nitrocellulose membranes rinsed using Smart Wash and the conventional washing method. The signals from individual slots were measured, plotted, and the S/N ratio was calculated. B. Simultaneous fluorescent detection using anti-β-actin and anti-His-tag antibodies on nitrocellulose membranes. Different amounts of cell lysates from AP-His-transfected HEK293 cells (1:2 serial dilutions starting from 6 μg/lane) were separated by SDS-PAGE, followed by immunoblot.

SUPPORTING MOVIE 1

A video demonstrating how to thoroughly rinse the membrane after antibody incubation.

SUPPORTING MOVIE 2

A video demonstrating how to set up the Smart Wash apparatus.

SUPPORTING MOVIE 3

A video of the Smart Wash operation.

Detailed Procedure for Smart Wash

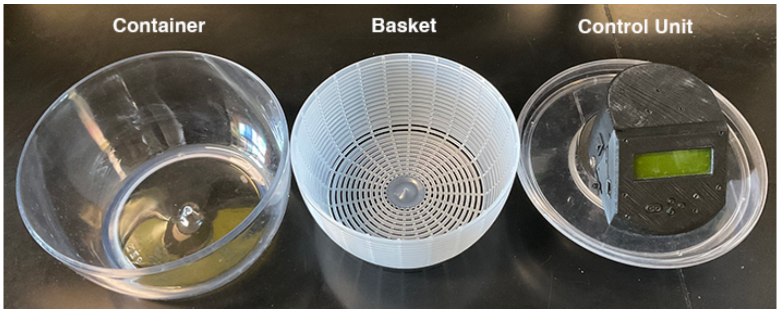

Materials

- PVDF membranes incubated with antibodies
- PBS-T (1.5 L)
- Deionized water
- Forceps
- Smart Wash device (container, basket, and control unit)
- A small container (11.5 x 8 cm)

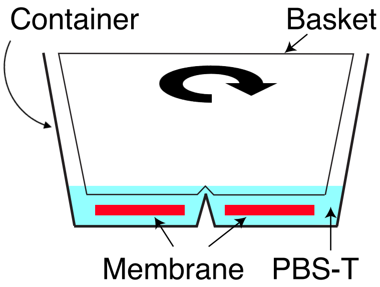

Procedures

1. Set up the standard rinsing conditions on the control unit.

Number of Cycles	4
Waiting Time (sec)	2
Duration for clockwise rotation (sec)	5
Speed for clockwise rotation	4
Duration for counterclockwise rotation (sec)	5
Speed for counterclockwise rotation	4

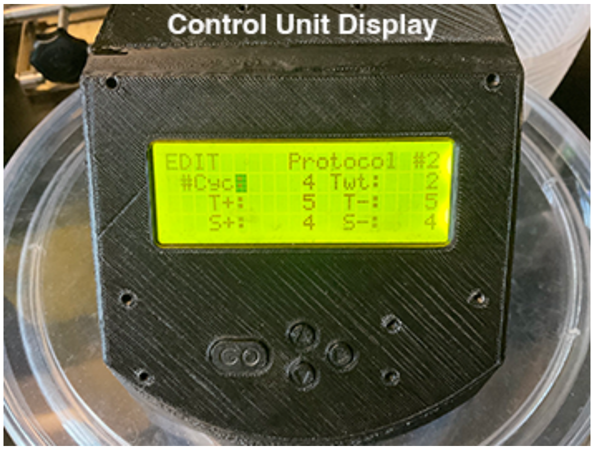

2. After antibody incubation, transfer the membrane to a small container containing 50 ml of PBS-T using forceps.
3. Rinse the membrane thoroughly with deionized water to remove most of the antibodies until the detergent-related bubbles disappear (Supporting Movie 1).
4. Add 200 ml of PBS-T to the Smart Wash container.
5. Transfer the membrane to the Smart Wash container using forceps. Place the basket in the container then place the control unit (Supporting Movie 2).
6. Start the rotation. It takes 65 sec to complete one wash.
7. Remove the control unit and basket from the container.

8. Transfer the membrane to a small container containing 50 ml of PBS-T. This is to prevent it from drying out.
9. Rinse the Smart Wash container and basket with tap water, then rinse further with deionized water.
10. Repeat Steps 4-9 twice.
11. After the final wash, proceed to acquire the image.

Note

- Up to 4 membranes (8 x 6.5 cm) can be rinsed simultaneously without drastic changes in the quality of the images.
- When the Smart Wash container and basket are dried out after use, higher background signals, especially with chemiluminescent detection are likely. Rinsing the Smart Wash unit immediately after each use is recommended.
- Fine tuning of the rinsing conditions may be required depending on the antibodies used in the study.
- When two membranes were incubated with two different antibodies and rinsed together using the Smart Wash, we observed cross-contamination, particularly with chemiluminescence detection.
